# Individual and community-level socioeconomic position and its association with adolescents experience of childhood sexual abuse: a multilevel analysis of six countries in Sub-Saharan Africa

**DOI:** 10.5249/jivr.v6i1.316

**Published:** 2014-01

**Authors:** Ismail Yahaya, Antonio Ponce de Leon, Olalekan A. Uthman, Joaquim Soares, Gloria Macassa

**Affiliations:** ^*a*^ Department of Public Health Sciences, Midsweden University, Sweden.; ^*b*^ Centre for Evidence-Based Global Health, Nigeria.; ^*c*^ Division of Social Medicine, Department of Public Health Sciences, Karolinska Institute, Sweden.; ^*d*^ Warwick-Centre for Applied Health Research and Delivery (WCAHRD), Division of Health Sciences, Warwick Medical School, The University of Warwick, Coventry, CV4 7AL, United Kingdom.; ^*e*^ Liverpool School of Tropical Medicine, International Health Group, Liverpool, Merseyside, UK.; ^*f*^Department of Occupational and Public Health Sciences, University of Gavle, Sweden.

**Keywords:** Childhood sexual abuse, Sexual violence, Sub-Saharan Africa, Socio-economic status, Neighborhoods, Health survey

## Abstract

**Background:**

Childhood sexual abuse (CSA) is a substantial global health and human rights problem and consequently a growing concern in sub-Saharan Africa. We examined the association between individual and community-level socioeconomic status (SES) and the likelihood of reporting CSA.

**Methods:**

We applied multiple multilevel logistic regression analysis on Demographic and Health Survey data for 6,351 female adolescents between the ages of 15 and 18 years from six countries in sub-Saharan Africa, between 2006 and 2008.

**Results:**

About 70% of the reported cases of CSA were between 14 and 17 years. Zambia had the highest proportion of reported cases of CSA (5.8%). At the individual and community level, we found that there was no association between CSA and socioeconomic position. This study provides evidence that the likelihood of reporting CSA cut across all individual SES as well as all community socioeconomic strata.

**Conclusions:**

We found no evidence of socioeconomic differentials in adolescents’ experience of CSA, suggesting that adolescents from the six countries studied experienced CSA regardless of their individual and community-level socioeconomic position. However, we found some evidence of geographical clustering, adolescents in the same community are subject to common contextual influences. Further studies are needed to explore possible effects of countries’ political, social, economic, legal, and cultural impact on childhood sexual abuse.

## Introduction

Childhood sexual abuse (CSA) against girls (defined as sexual violence experienced by female children below the age of 18 years) is a substantial global health and human-rights problem and a growing concern in sub-Saharan Africa.^1^ The World Health Organisation (WHO) Global School-based Student Health Survey (SHS) documented the widespread nature of sexual abuse in children,^2^ with lifetime prevalence of sexual abuse among students 13-15 years of age in the five countries surveyed, ranged from 9% to 33%. In a review of population based studies, Pereda and colleagues found that 0% to 53% of women reported that they had experienced CSA.^3^ CSA is also associated with physical, social and psychological effects on young women.^4-12^ A troubling aspect of CSA is underreporting of cases. In SSA including the six countries in this study, most researchers believe that statistics of CSA under-represent the actual number of victims. The embarrassment, shame or fear of being blamed and a desire to keep the abuse secret make disclosure uncommon.^13,14^ Others stay silent for fear of provoking further violence, or insensitive interventions which could make their overall situation worse. 

Individual based socioeconomic position has been documented to be a contributing factor to sexual violence.^15^ Higher socioeconomic status (SES) levels among women have generally been found to be protective factors against the risk of sexual violence towards women.^15^ In contrast, most studies on CSA are not associated with SES. The risk factors identified for CSA in preadolescents (before 10 years) and early adolescents (10 to 14 years) include having a stepfather, living without a natural parent, having an impaired mother, poor parenting, or witnessing family conflict.^10,16^ Such individual level factors under examination are limited in their scope and do not address how CSA is influenced by wider social structural forces. Recently, community-level factors have been the focus of attention when considering risk factors for violence. The association between area based socioeconomic indicators and health outcomes have been documented in recent studies.^17,18^ Although the mechanisms by which area based SES affects health are not clear, it has been suggested that community SES could influence health behaviours and health related beliefs of their residents, independent of their personal SES.^19,20^

Strong evidence exists that contextual factors are important in determining levels of sexual violence across groups.^21^ Studies from developing and developed countries show that community-level measures of SES have significant effects on the risk of sexual violence. Previous research has focused predominantly on other forms of sexual violence especially intimate partner violence. To date, there are no studies that have investigated the role of socioeconomic indicators and community socioeconomic conditions simultaneously on CSA in sub-Saharan Africa. Understanding social factors such as SES, which are likely fundamental causes of health outcomes, are necessary to help adopt broad-based societal interventions that could produce substantial health benefits.^22^ Other factors which can increase the vulnerability to sexual violence (especially due to social, economic and political crises) include wars, political strife, natural and manmade disasters, as they disrupt the formal and informal protection mechanisms of families, communities and the states. However, such factors are not dealt with in this study.

**Conceptual Framework**

In this study, we drew on the elements of a socio-ecological model to examine the associations between neighbourhood factors and CSA.^4,23^ The socio-ecological model recognizes the interwoven relationship that exists between the individual, relationship, community and societal factors.^12^ The model explores the relationship between individual and contextual factors and considers violence as the product of multiple levels of influence on behaviour. The more homogenous the health of people within a neighbourhood is (as compared with the health of people from different neighbourhoods), the more probable it is that the determinants of individual health are directly related to the contextual environment of the neighbourhood and/or that social processes of geographical segregation are taking place.^24^ The socio-ecological model has been used extensively to better understand violence and the effect of potential prevention strategies.^25^ The benefit of the socio-ecological model lies in its capacity to consider, in a systematic way, the factors that influence health behaviours; in this case those factors that put people at risk of experiencing or perpetrating violence.^12^ Community measures of poverty have been found to have the greatest explanatory power among socio-ecological theory variables.^26^ Community poverty weakens social network and the capacity to control the behaviour of people and hence increase the likelihood of reporting CSA.^27^

We also adopted the concept of social control theory which postulates that individuals are inherently inclined to become deviant as their ties to the “conventional order” within society becomes or is broken.^28^ According to the theory, strong social bond to social institutions promotes conformity to conventional norms, and hence individuals who possess weak or broken social bonds to conventional institutions are more likely to engage in deviant behaviour^28^ in this case CSA. According to differential association theory, negative behaviour is learned through interaction with other deviant individuals and these interactions are formed through social and cultural transmission.^29^ Both social control theory and differential association theory show the importance of familial cohesion, parental stressors, and neighbourhood environment in the development of pro-social behaviour in deterring negative behaviour.^30^

To the best of our knowledge, no studies exist that have examined the contributions of individual and contextual factors associated with CSA in sub-Saharan Africa. Thus, the overarching aim of this study was to fill this research gap and contribute to the existing literature on CSA. The specific objectives were (1) to examine individual and contextual factors associated with CSA and (2) to examine whether there is significant community level variation in reported CSA; whether people living in the same community share similar probability of reporting CSA.

## Methods

The data reported here were from the Demographic and Health Surveys (DHS) conducted in six countries in sub-Saharan Africa (Ghana, Liberia, Nigeria, Uganda, Zambia and Zimbabwe) between 2006 and 2008. The six countries were chosen because they met the selection criteria of recent surveys during the past 10 years and because of the availability of data sets on sexual violence.

DHS surveys were designed to collect good quality, nationally representative data on demographic and health indicators of women and members of their households. In general, the surveys were well conducted with a high response rate (average of 96%). Methods and data collection procedures have been published elsewhere.^31^ Briefly, the survey applied utilised a two-stage cluster sampling design. The first stage involved taking up enumeration areas from Census files while in the second stage, a sample of households was drawn from an updated list of households within each enumeration area. Every survey was stratified by urban and rural status and additionally by country-specific geographic or administrative regions. A standardised questionnaire was administered by interviewers to all the female participants aged between 15 and 49 years in the selected households. To ensure standardisation and comparability across sites and time, DHS surveys employ intense interviewer training, standardised measurement tools and techniques, identical core questionnaires and instrument pretesting.^32^ The number of women included in the six DHS ranged from 4,916 in Ghana to 33,885 in Nigeria. The DHS survey was implemented by respective national implementing agencies with technical assistance from ICF Macro International Inc (Calverton, MD).

**Outcome Variable**

To be included in the analysis, the respondents were required to meet the following two criteria: (1) they must be eighteen years or younger and (2) they must be a permanent resident at the place where the interview was conducted. For this study, CSA was defined as sexual violence on or before the age of 18 years. To assess if participants were sexually abused in childhood, all eligible women were asked the following questions: “At any time in your life, as a child or as an adult, has anyone forced you in any way to have sexual intercourse or perform any other sexual acts?” The two possible outcomes for the questions were “yes” or “no”. Respondents who answered yes were then asked questions about the age at which the abuse first occurred and the identity of the person who committed the act. Respondents who answered yes and in cases where the violence occurred when they were under the age of 18 years, were considered as suitable cases of CSA and coded as “1”, while those who responded no or if the abuse occurred after the age of 18 years, formed the other group of the dichotomy and were coded “0”. All the women who did not answer the question were excluded from the survey. 

**Determinants Variables**

**Individual Level Factors**

The main independent variables of interests at the individual level were wealth status and level of education. The wealth index was constructed using easy-to-collect data on the household's ownership of selected assets, such as televisions and cars, dwelling characteristics such as flooring materials, type of drinking water sources, toilet facilities and other characteristics that are related to wealth status. They were then assigned a weight or factor score generated through principal component analysis.^33^ The weighted scores were divided into five quintiles for the analytic models (poorest, poorer, middle, richer and richest).^34^ The level of education of the participants was categorized into: no formal education or educated. 

**Community-level Factors**

Within the DHS, communities were defined as the primary sampling unit (PSU). The PSU was based on the most recent sampling frame for each country as defined by census enumeration blocks. We included two community level factors: community SES and place of residence. Community SES was an index constructed from three variables using principal component analysis. The variables are the proportion of respondents: with no education (illiterate), unemployed and living below the poverty level (asset index below 20% poorest quintile). A standardised score with mean 0 and standard deviation 1 was generated from this index, which we divided into five quintiles (quintiles 1 to 5). Quintile 5 represented highest SEP while quintile 1 represented lowest SEP. Place of residence was categorised into either rural or urban residence. 

**Control Variable**

Country of residence was also included as a categorical variable. The country was included as a partial control variable to control for the effects of unknown factors due to potential differences across the six countries.

**Ethics**

The surveys were approved by the ICF Macro’s Ethics Committee, USA and the National Ethics Committee in the Ministry of Health of the respective countries. Informed consent was obtained from participants before the collection of all data.

**Statistical Analysis**

In the descriptive statistics, frequency tabulations were conducted to describe the distribution of correspondents. The key variables were expressed as percentages. Given the hierarchical structure of the sample and the binary outcome, a logistic multilevel modelling approach was adopted. We specified a two-level model for binary response reporting CSA or not, for adolescents (level 1) living in a community (level 2).

Three models were fitted. In the first model, an empty model, no explanatory variable was included. This model was focused on decomposing total variance into its individual and community components. In the second model, only control variable was included. The third model (full model) included control, individual and neighbourhood variables.

The results of the fixed effect model (measures of association) were expressed as odds ratios (ORs) with 95% confidence intervals (CI). The results of the random-intercept models (measures of variation) were presented as variance partition coefficient (VPC) and proportional change in variance (PCV). The VPC was calculated by the linear threshold (latent variable method) according to the formula used by Snijders^35^ as follows:

$$ VPC= \frac{V_{c} {V_{c} + (\frac{{\pi}^2}{3})}} $$

where V_c_ = community level variance.

We calculated the PCV as follows:

$$ PCV= \frac{({V}_{a} - {V}_{b}) {{V}_{a}}} \cdot 100 $$

where V_a_ = variance of the empty model, and V_b_ = variance of the model with more terms.

MLwin software, version 2.10^36^ was used for the analysis. The statistical significance of covariates was calculated using Wald’s test. All significance tests were two-sided and statistical significance was defined at 5% level.

## Results

**Sample Characteristics**

**Figure 1:**
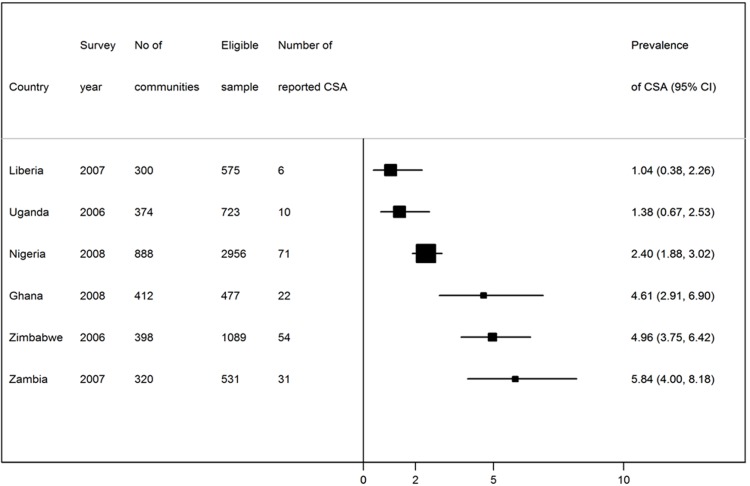
Description of Demographic and Health Surveys data 2006-2008 in sub-Saharan Africa by country, survey year, communities sampled, eligible sample and reported childhood sexual abuse (CSA)

**Figure 2:**
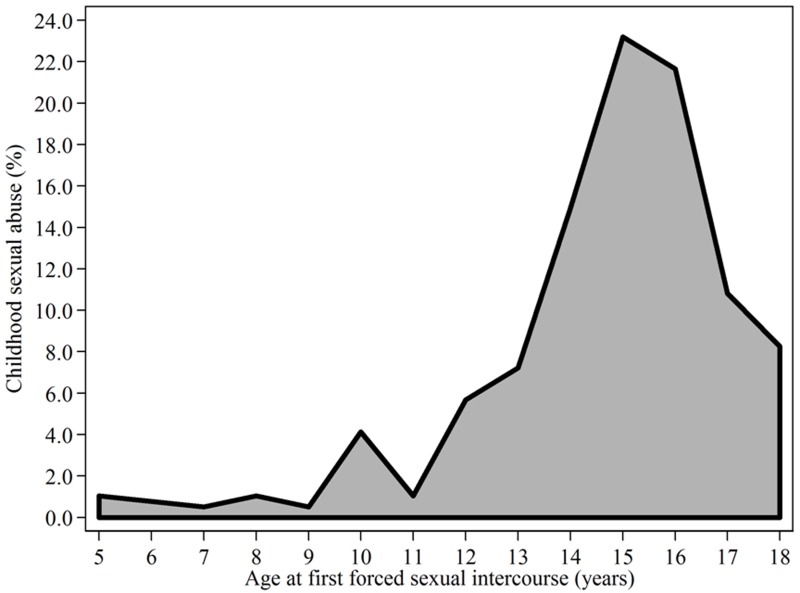
Age at which first forced sexual intercourse occurred, as reported by the adolescents

The countries, the survey year, and the eligible sample are shown in Figure 1. The number of adolescents who were permanent residents in the area at the time of the survey and thus included in the study was 477 in Ghana and 2,956 in Nigeria. The number of communities sampled ranged from as few as 300 in Liberia to as many as 888 in Nigeria. The percentage of adolescents that had experienced CSA ranged from 1.04% in Liberia to 5.84% in Zambia. The youngest age of exposure to CSA was 5 years old (Figure 2). As shown in Figure 2, the most common age at which respondents experienced CSA was between 14 and 17 years of age, with 70% of the reported cases of CSA occurring at this age group. The descriptive statistics of respondents are presented in Table 1. A total of 6,351 adolescents were analysed in this study. About one-fifth (12%) of the respondents were educated. Almost half of the respondents (46%) were sampled from Nigeria.

**Table 1:** Percentage distribution of selected characteristics

Variable	Total number (%)
Individual level	(n=6351)
Educated	
Yes	763 (12.0)
No	5,588 (88.0)
Wealth index	
Poorest	1,257 (19.8)
Poorer	1,313 (20.7)
Middle	1,410 (22.0)
Richer	1,315 (20.7)
Richest	1,056 (16.6)
Community Level	
Place of residence	
Urban	4,481 (70.6)
Rural	1,870 (29.4)
Community SES	
Quintile 1	1,569 (24.7)
Quintile 2	1,451 (22.8)
Quintile 3	1,229 (19.4)
Quintile 4	1,081 (17.0)
Quintile 5	1,021 (16.1)

**Measures of Variability (random intercept models)**

The result of the random-intercept model is shown in Table 2. The empty model (null model) shows that there was a significant variation in the odds of reporting CSA across the communities (α=0. 926, p=0. 036). The intra-community correlation coefficient as implied by the intercept component variance specified that 22% of the variation in CSA could be attributed to the community level factors. As judged by a proportional change in variance, almost half (46%) of the variance in the odds of reporting CSA across communities was explained by country domain variable. After adjusting for all the variables in the full model (Model 3), about 70% of the variance in the odds of reporting CSA across communities was explained by all the variables included. The variations across communities became not statistically significant after controlling for control-variable in Model 2 and all variables in Model 3.

**Table 2:** Fixed- and random-intercept parts of multilevel logistic regression of childhood sexual abuse

Measures of association	Model 1^a^ OR (95% CI)	Model 2^b^ OR (95% CI)	Model 3^c^ OR (95% CI)
Control-variable			
Country			
Ghana		0.20 (0.08 – 0.48)***	0.20 (0.08 – 0.47)***
Liberia		0.03 (0.01 - 0.20)***	0.03 (0.01 - 0.22)**
Nigeria		0.40 (0.26 - 0.62)***	0.43 (0.28 - 0.68)***
Uganda		0.13 (0.05 - 0.32)***	0.12 (0.05 - 0.30)***
Zambia		1 (reference)	1 (reference)
Zimbabwe		0.19 (0.10 - 0.36)***	0.17 (0.09 - 0.34)***
Individual-level			
No education			0.36 (0.16 – 0.81)*
Wealth index			
Poorest			1.06 (0.46 – 2.44)
Poorer			0.84 (0.39 – 1.79)
Middle			0.83 (0.42 – 1.67)
Richer			0.88 (0.46 – 1.65)
Richest			1 (reference)
Community-level			
Urban (vs. rural)			0.67 (0.40 – 1.15)
Community SES*			
Quintile 1 (least)			1 (reference)
Quintile 2			0.96 (0.56 – 1.64)
Quintile 3			1.05 (0.59 – 1.87)
Quintile 4			1.20 (0.62 – 2.30)
Quintile 5 (most)			1.04 (0.46 – 2.33)
Measures of variation			
Community-level			
Variance (SE)	0.926 (0.442)*	0.505 (0.370)	0.274 (0.345)
ICC (%)	21.9	13.3	7.7
Explained variation (%)	reference	45.5	70.4

a Model 1 – Empty model – no explanatory variables b Model 2 – Adjusted for control variable (country as a fixed-effect) c Model 3 – Adjusted for control-, individual- and community-level factors * Community-SES – community socioeconomic disadvantages, quintile 1 – least disadvantaged, quintile 5 – most disadvantaged Abbreviations: OR – odds ratio, CI – confidence intervals, SE- standard error, ICC – intra-community correlation

**Measures of Associations (fixed effects)**

The results of fitting the model including control, individual and community-level variables are also displayed in Table 2. Only country dummies and level education were statistically significantly associated with CSA. Respondents with no education were 64% less likely to have reported CSA than those with formal education (odds ratio [OR] = 0.36, 95% confidence interval [CI] 0.16 to 0.81). Compared with Zambia, residents in the other five countries were less likely to have reported CSA: Ghana (OR 0.19, 95% CI 0.09 to 0.47), Liberia (OR 0.03, 95% CI 0.01 to 0.22), Nigeria (OR 0.43, 95% CI 0.28 to 0.68), Uganda (OR 0.12, 95% CI 0.05 to 0.30) and Zimbabwe (OR 0.17, 95% CI 0.09 to 0.34). 

## Discussion

The present study examined the way in which individual and community level SES influenced the history of sexual abuse in childhood. The causes of CSA are many and complex, and there are numerous ideas that have been proposed to help understand this phenomenon. Research has focussed on the effect of the family, the family environment, poverty, parental stress and other factors on CSA. The present study expands on the current literature in that it examined the way in which SES influenced the history of sexual abuse in childhood. Using the social ecological model, we hypothesised that history of CSA would be associated with community socioeconomic status (SES). The results of the present study corroborates those of previous studies on CSA that found no evidence of association between SES and history of CSA ^10,37,38^ and between area of residence (rural/urban) and history of CSA.^39,40^ CSA transcends across all SES at the individual level. More importantly, the findings uncover new evidence that risk of CSA cuts across all strata of community socioeconomic context. We found no evidence that CSA was associated with community-level SES. Though, these variables were not statistically significant, it should not imply that they are not important from public health perspective. This suggests that there may be lack of evidence of socioeconomic inequality in the occurrence of CSA; regardless of the household socioeconomic status, children had equal exposure to CSA. One possible explanation could be that we did not have large enough sample size to reach statistically significant level.

Counter intuitively, we found that uneducated women were less likely to have reported CSA. The higher prevalence of abuse reported in educated girls compared to non-educated girls was unexpected. This finding requires further investigations, as high level of education is not a common factor for CSA.^37^ A possible contributory factor may be the high rate of reported sexual violence in African schools.^13^ Although, most of the perpetrators of sexual abuse are either family members or those known to the victim, most cases of this maltreatment still takes place either on the way to school, in schools or on the way back from schools.^1^

Importantly, the results suggest that adolescents living in the same community tended to report CSA. It is possible that adolescents in the same community were subjected to common contextual influences.^24^ However, the preponderance of the variance was explained by individual, community-level SES. Almost half of the variance in the community was explained by unmeasured country effects. Similarly, variations across communities proved to be not statistically significant after controlling for the effects of unmeasured control factors. This suggests that the likelihood of reported CSA may be due to the unmeasured country-level effects. It is possible that the community-level variance was due to shared social norms. The full model was able to explain most of the observed variations. The findings have important implications for targeting policy as well as the exploration of factors not included in the model that could explain the remaining unexplained variation.

Although this study used socio ecological framework to examine how individual and community level SES influence CSA, it is important to highlight concerns with reporting CSA. Children born to victims of CSA are at increased risks of being risks themselves. Because these children live in a violent environment, they are at risks either at the hands of their own caregivers or from the deleterious consequences of having a caregiver who suffers from the emotional, psychiatric or physical sequelae of her own childhood abuse.^41^ Ayala and colleagues also points out that domestic violence increases the probability of sexual abuse within the family.^42^ The abuse of such children in the hands of their parents has been linked to either the parents being neglectful or through recreation of environmental conditions in which abuse was allowed to persist across generations.^43^ Such situation has led to the notion that abusing parents were themselves abused and that abused and neglected children would become tomorrow’s perpetrators of family violence.^44^ A vast proportion of sexual violence takes place within the victim’s immediate environment with majority of the perpetrators known to the victims but yet are not reported. Most sexually abused children do not tell anyone they were abused, even when directly asked by parents or other authority figures. The stigma associated with being a victim of sexual assault makes it difficult for victims or even their parents to report suspected cases. Within a family, cases of CSA may go unreported by a spouse to avoid marital separation, divorce, loss of friends, loss of job and loss of income.^45^ The secrecy and collusion within the family serves as a barrier for any family member to report the abuse. In addition to this, there are strong norms against informing on one’s family members.^46^

**Study Limitations and Strengths**

Despite the contribution our study makes to the existing literature on neighbourhood effects, we are aware of certain limitations. Primarily, the variables available were restricted because the data from this study was drawn from national surveys. Although we were able to construct CSA variable and utilise some socio-demographic factors, other predictors of CSA such as family stability and marital conflict, parenting and parent-child relationships and parental adjustment were not available in the survey and so could not be included in the analysis. Further, some of these variables might have explained some associations between community and CSA. In addition, almost all of the samples included in the model were from Nigeria. It is possible that the associations may be influenced by the large number of adolescents from Nigeria. However, we found that not only Nigeria’s country dummy was associated with the likelihood of reporting CSA. As this is a cross sectional study, it is difficult to assess the direction of causality from its findings. Data was collected through self-report and because of the sensitive nature of the questions being asked, there is the likelihood that some respondents might not have disclosed their past experience(s). Therefore, it is unlikely that an exact account of the exposure to the CSA will be available. It is also feasible that the cases of CSA are underreported because of the stigma associated with sexual violence. Finally, our survey does not have data on household income. We utilised asset-based wealth index which is a proxy indicator of household economic status. 

Despite these limitations, the strengths of the study are important. To the best of our knowledge, this is one of the first studies to examine the association between community SES and CSA applying a multilevel approach. It is also a large, population based study from six countries in sub-Saharan Africa with high response rates. It is a nationally representative sample with similar indicators used across regions and countries, making it possible for numerical values to be compared across the sites. More so, the data obtained from DHS was widely perceived to be of high quality based on sound sampling methodology and adherence to ethical standards of data collection including violence data. ^32^ About 70 % of the community variations in the reported CSA were accounted for by individual and community contextual characteristics, signifying that the models were effective in predicting the risk of CSA. Similarly, we were able to limit and avoid cohort effect and recall bias. We included only adolescents in the model. By including only respondents aged 18 or younger, we were able to improve timeliness of information gathering, such that the interval between the event (CSA) and the interview (the recall period) is as short as possible, thus reducing non-differential recall bias. Similarly, by including only permanent residents at the time of the interview, we were able to prevent cohort effect - that is, variations in the characteristics of the community over time among respondents who are defined by some common life experience or shared temporal experience.

## Conclusion

This study provides evidence that likelihood of reporting CSA cuts across all individual SES as well as all community socioeconomic strata. Adolescents in the same community may be subjected to common contextual influences. Further studies are needed to explore possible effects of countries’ political, social, economic, legal and cultural factors on CSA. 
